# Influence of Pulsed He–Ne Laser Irradiation on the Red Blood Cell Interaction Studied by Optical Tweezers

**DOI:** 10.3390/mi10120853

**Published:** 2019-12-05

**Authors:** Ruixue Zhu, Tatiana Avsievich, Alexander Bykov, Alexey Popov, Igor Meglinski

**Affiliations:** 1 Optoelectronics and Measurement Techniques Laboratory, University of Oulu, 90570 Oulu, Finland; ruixue.zhu@oulu.fi (R.Z.); tatiana.avsievich@oulu.fi (T.A.); alexander.bykov@oulu.fi (A.B.); alexey.popov@oulu.fi (A.P.); 2 Interdisciplinary Laboratory of Biophotonics, National Research Tomsk State University, 634050 Tomsk, Russia; 3 Institute of Engineering Physics for Biomedicine (PhysBio), National Research Nuclear University (MEPhI), 115409 Moscow, Russia; 4 Aston Institute of Materials Research, School of Engineering and Applied Science, Aston University, Birmingham B4 7ET, UK; 5 School of Life and Health Sciences, Aston University, Birmingham B4 7ET, UK

**Keywords:** optical tweezers, red blood cells (RBCs), RBCs interactions, helium–neon laser, laser irradiation

## Abstract

Optical Tweezers (OT), as a revolutionary innovation in laser physics, has been extremely useful in studying cell interaction dynamics at a single-cell level. The reversible aggregation process of red blood cells (RBCs) has an important influence on blood rheological properties, but the underlying mechanism has not been fully understood. The regulating effects of low-level laser irradiation on blood rheological properties have been reported. However, the influence of pulsed laser irradiation, and the origin of laser irradiation effects on the interaction between RBCs remain unclear. In this study, RBC interaction was assessed in detail with OT. The effects of both continuous and pulsed low-level He–Ne laser irradiation on RBC aggregation was investigated within a short irradiation period (up to 300 s). The results indicate stronger intercellular interaction between RBCs in the enforced disaggregation process, and both the cell contact time and the initial contact area between two RBCs showed an impact on the measured disaggregation force. Meanwhile, the RBC aggregation force that was independent to measurement conditions decreased after a short time of pulsed He–Ne laser irradiation. These results provide new insights into the understanding of the RBC interaction mechanism and laser irradiation effects on blood properties.

## 1. Introduction

Human red blood cells (RBCs), or erythrocytes, can form face-to-face two-dimensional rouleaux, which can branch into three-dimensional structures by side-to-side or side-to-end contact of cells in aqueous suspension of large proteins or polymers. The spontaneous process of RBCs clumping is known as aggregation, whereas the enforced rouleaux dispersion by high-shear conditions is referred to as disaggregation. RBC (dis)aggregation highly depends on suspension properties including the concentration of fibrous biomolecules, the molecular weight of neutral polymers, and the antagonistic or synergetic effects between different proteins [1,2]. The (dis)aggregation behavior also relies on RBC intrinsic properties, such as cell age, shape, other donor-specific factors [3], membrane electrical properties (zeta potential) [4], RBC deformability [5], and properties of the glycocalyx on the outer surface of the cell membrane [6]. Measurements and analysis of RBCs mutual interaction have long been a hot topic of real-life significance for several reasons. First, RBC interaction is one of the most important hemorheological determinants that affects blood flow rheology, functional capillary density, organ perfusion, and distribution of other cells (e.g., leucocytes and platelets margination) Ref [7]. Secondly, the speed of blood aggregation is critical for hemostasis to control hemorrhage and maintain the circulating blood within the intravascular space when external or internal bleeding occurs [8]. Moreover, the degree of RBC aggregation is closely related to the pathological status of certain diseases (e.g., inflammation, cardiovascular embolism, and rheumatoid arthritis), and may serve as an early indicator for diagnosis [9]. Thus, the better understanding of RBC interaction mechanism and investigation of potential methods to regulate this process are important for the future blood microcirculation monitoring and therapy.

Typical RBC aggregation analyses are based on parameters indicating the extent and rate of rouleaux formation calculated from the time course required for RBC suspension to approach a final stable sedimentation state [10], individual cell-cell interaction dynamics cannot be revealed in detail. The development of single-cell level methods such as micropipette aspiration technique (MAT), atomic force microscopy (AFM), and scanning electron microscopy (SEM), has promoted the cell biology study and helped unravel the different structures and function of living cells on a microscopic and molecular level [11,12]. Among these methods is the Optical Tweezers (OT), a significant achievement of laser physics with the ability to non-invasively trap, manipulate and displace a living cell or part of it with highly accurate positioning that has deepened the investigation of intercellular interactions [13,14]. Since the first application of OT to RBC interaction study in 1997 [15], numerous significant results, such as quantified RBC parameters (e.g., membrane viscosity and zeta potential) [16] and the effects of heparin and tranexamic acid on the efficiency of RBC aggregation [17], have been obtained. Furthermore, based on the RBC aggregation behavior measured by OT, a sensitive monitoring method for systemic lupus erythematosus (SLE) and its response to drug therapies has been established [18]. The 2018 Nobel Prize in physics was awarded to the pioneer Dr Arthur Ashkin ”for the optical tweezers and their application to biological systems”. Unquestionably, the unique advantages of OT in micromanipulation as well as in precision measurement of ultra-low forces at piconewton ($10^{-12}$ N) level have made it an essential tool in the field of biological cytology.

He–Ne laser sources are widely used in clinical diagnosis and treatments including accelerating wound healing of soft tissue, regulating metabolic activities, and aiding the treatment for acute cerebral infarction [19,20]. Low-level laser irradiation has great potentiality of regulating blood rheological properties (e.g., blood viscosity and electrophoretic mobility) and RBCs interaction in blood samples with abnormally high erythrocyte sedimentation rate (ESR) [21]. However, most studies were based on statistical analysis by traditional instruments such as Wintrobe tube for ESR, red cell deformability meter, and electrophoretic meter for zeta potential [22,23]. The origin of laser irradiation effects on blood properties, especially on RBC aggregation behavior is not clearly understood. The absorption of irradiated energy by hemoglobin is assumed to be the main mechanism as greater influence was observed by laser wavelength closer to the hemoglobin absorption band (400–600 nm) with a longer irradiation time (up to 130 min) [21,24].

In our study, OT was used to thoroughly assess RBC (dis)aggregation properties and investigate short-time laser irradiation effects on RBC interaction at a single-cell level. The results not only clarified the applicability of the two co-existent models to different RBC interaction processes, but also indicated the dependence of the disaggregation force on both the cell interaction time and initial contact area between two RBCs. Furthermore, by studying low-level laser irradiation effects on RBC aggregation within a short irradiation period (up to 300 s), an attempt was made to explore other possible laser-cell interaction mechanism rather than thermal absorption, which is predominant in long-time irradiation. The results showed that low-level He–Ne laser with appropriate pulse frequency has the potentiality to reduce the RBC aggregation force in a short irradiation time (120 s).

## 2. Materials and Methods

The experimental samples were highly diluted RBCs suspensions in platelet-low autologous plasma (hematocrit<1%) donated by a single healthy donor (female, age 26) with oral consent and under ethical permission (Finnish Red Cross, No. 11/2019) to avoid introducing donor-specific differences to the measurements. The fresh RBCs were obtained by centrifuging diluted suspensions of fingertip-prick blood drops in phosphate buffer saline (PBS) at 6500 RPM (4732 g) for 10 min every time before experiments. The platelet-low plasma was obtained by double washing of whole blood samples obtained by venipuncture at a Nordlab clinic (Oulu, Finland) under the same condition, and was stored in a fridge for up to one month. The sample chamber with capacity of 20–30 μL liquid samples was composed of a microscope slide and a cover glass that were bonded by double-sided tape (100 μm thick), and was sealed by Vaseline after sample injection. All measurements were performed within 3 h after sample preparation at room temperature ( 23 ${}^{\circ }C$).

**Figure 1:**
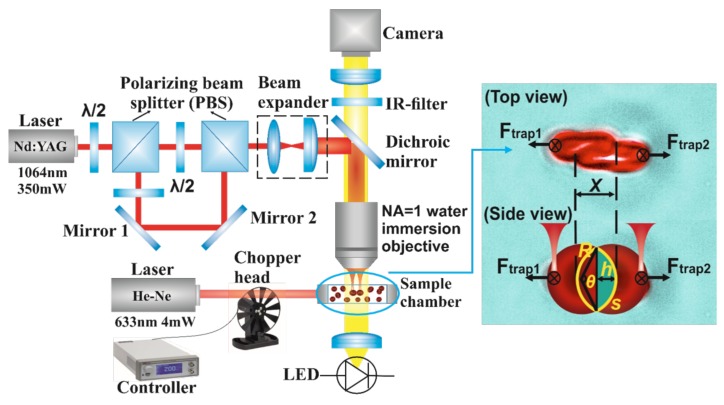
Schematic layout of the double-channel optical tweezers system combined with a chopper-modulated pulsed laser irradiation module for studying RBC interaction and laser irradiation effects. Two orthogonally polarized beams separated from one infrared laser source were focused simultaneously to form two independent trapping channels. He–Ne laser modulated by an optical chopper system was used to irradiate the trapped RBC rouleau in the sample chamber. Top-view image was captured by the CMOS camera in transmission mode and the interaction area between the two RBCs was calculated according to the side view geometries.

Double-beam polarization-based optical tweezers (OT) was used as the main experimental tool to investigate cell interaction dynamics. The basic requirement is the strong focusing of the laser beam. The trapping phenomenon can be explained by momentum transfers involved in the interaction between the strong focused laser beam and the micro-sized particle (RBC) adjacent to the beam focus [25]. The construction of the in-house-made two-channel OT system combined with a chopper-modulated pulsed laser irradiation module is shown in Figure 1. Laser beam from an infrared single-mode Nd:YAG laser source (1064 nm, 350 mW, ILML3IF-300 Leadlight Technology, Taiwan) was divided into two orthogonally polarized continuous-wave beams that were then simultaneously focused by a large numerical aperture water immersion objective (lLUMPlanFl 100×/1.00 W, Olympus, Tokyo, Japan) to form two independent trapping channels in the focal plane within the sample chamber. One trapping beam was located in the center of the observation plane, and the position of the second trap was adjustable by stirring mirrors 1 and 2. The trapping power and the power ratio between the two channels were controlled through rotating half-wave plates. The orthogonal polarization states were adopted to reduce possible interference between the two beams, and the trapped cells were observed to align along the polarization direction within an optical trap. A beam expander was used to widen the trapping beams to fill the back aperture of the objective to achieve the maximum trapping efficiency. The observation system was made up of a white LED-illuminating source, a dichroic mirror, an IR-filter, and a CMOS camera (lPixelink PL-B621M, Ottawa, Ontario, Canada). The laser irradiation module consists of a He–Ne laser source (633 nm, 4 mW, beam diameter 0.48 mm, 1507P-0, JDS Uniphase, USA) and an optical chopper system (MC2000B, Thorlabs, USA) was used to irradiate the trapped RBC rouleau in the sample chamber. As shown in Figure 1, the linear overlapping distance *x* between the two trapped RBCs can be measured from the captured top-view image, and the corresponding interaction area *A*, which is enclosed by two arcs *s* (marked in yellow in the side view image), can be calculated as the sum of two segments using the following equation:

(1) $$A=R^{2}\;\cdot \;\left(\theta -\sin\theta \right),$$

where *R* is the radius of the RBC and θ is the central angle of the arc:

(2) $$\theta =2\;\cdot \;\arccos\left(\frac{R-h}{h}\right)=2\;\cdot \;\arccos\left(\frac{R-x/2}{x/2}\right),$$

where *h* is the height of the arc (the perpendicular from the midpoint of the arc’s chord to the arc itself). If the interaction force $F_{inter}$ is known under this overlapping area *A*, the corresponding interaction energy density *U* between the two RBCs can be estimated by the following equation:

(3) $$U=\frac{F_{inter}\;\cdot \;2h}{A}.$$

To quantify the optical trapping force applied to RBCs, force calibration was performed based on the force equilibrium between the optical trapping force and the viscous friction force exerted on a trapped RBC by a counterflow with a known velocity. In our case, blood plasma was the trapping environment, and the flow was generated by the lateral movement of the sample chamber attached to a XYZ-stage. The optical trapping force is linearly related to the trapping power, whereas the viscous friction force $F_{v}$ is linearly related to the flow velocity *v*, as described by Stokes’ law [26]:

(4) $$F_{v}=6\pi \eta RvK,$$

where η (1.30 mPas) is viscosity coefficient of the fluid (blood plasma) [27]; *R* (2.7 μm) is an effective radius of the RBC; *K* (1.36) is a correction factor for ellipsoid. The flow velocity was calculated from the video captured during the measurement. With a given trapping power, the velocity of the flow was slowly increased until the trapping force was matched by the viscous friction force and the RBC escaped from the trap.

## 3. Results

### 3.1. Trapping Force Calibration

**Figure 2:**
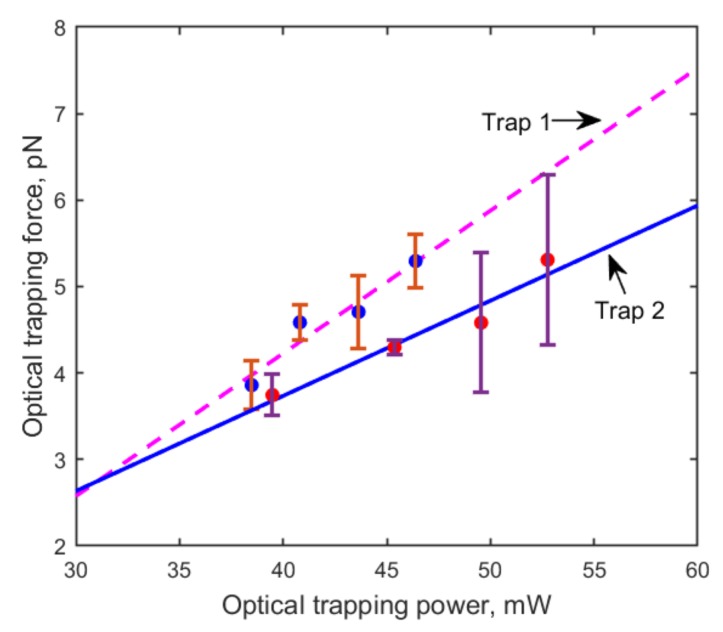
Trapping force calibration for the central beam (trap 1: pink dashed line) and for the movable beam (trap 2: black solid line). At least six measurements were performed with each trapping power. The trapping force in trap 2 is slightly weaker than in trap 1.

The linear relationship between the optical trapping force and trapping power for each of the two trapping channels is shown in Figure 2. It can be clearly seen that the trapping force of the movable beam (trap 2) is slightly weaker than that of the central beam (trap 1). This was also manifested by the experimental phenomenon that RBCs escaped first from the movable trap. Therefore, the linear fitting equation of the movable channel $F_{trap2}=0.11\times P-0.67$, where *P* is the optical trapping power, was used to calculate the optical trapping force. By measuring the laser power after the objective, the attenuation coefficient of the objective was calculated to be about 47%. The trapping efficiency *Q* was about 0.05, according to the following equation [28]:

(5) $$Q=\frac{c\;\cdot \;F_{trap}}{P_{e}\;\cdot \;n_{m}},$$

where *c* is light propagation speed in vacuum, $P_{e}$ is the effective trapping power, and $n_{m}$ is the refractive index of the trapping medium.

### 3.2. Interaction Energy Density of RBCs during Aggregation and Disaggregation Process

**Figure 3:**
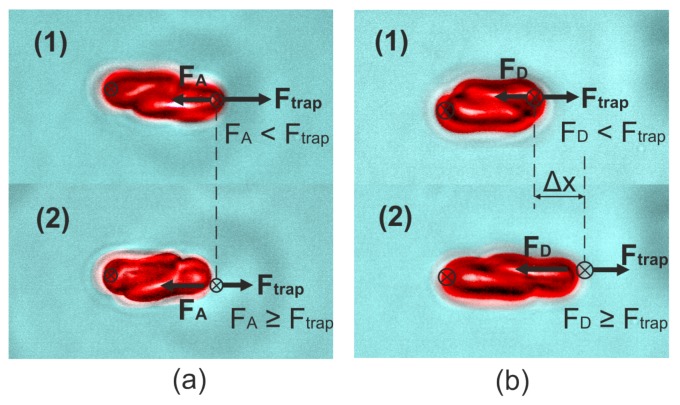
(**a**) Illustration of RBC aggregation force measurement. The position of the second trap was kept still while the trapping power was slowly decreased until the RBC escaped the trap; (**b**) Illustration of RBC disaggregation force measurement. The position of the second trap was slowly moved towards the direction to separate the two RBCs with a certain trapping power until the RBC escaped the trap. The small circles with crosses indicate the positions of the traps. $F_{trap}$ indicates the trapping force, $F_{A}$ and $F_{D}$ indicate aggregation and disaggregation force respectively.

With the two-channel OT system, the intercellular forces between two RBCs in rouleau formation (aggregation) and destruction (disaggregation) were measured separately, with the interaction energy density calculated for each process. For aggregation measurement as shown in Figure 3a, two individual RBCs were separately captured and lifted to a height of 40 μm from the bottom of the sample chamber by two trapping beams simultaneously, and then a linear contact between the two cells was formed under the control of OT. After 40 s interaction, the aggregation force ($F_{A}$) was measured as the minimum optical force applied to stop the two cells from clumping together by slowly decreasing the trapping power while keeping the contact area constant.

**Figure 4:**
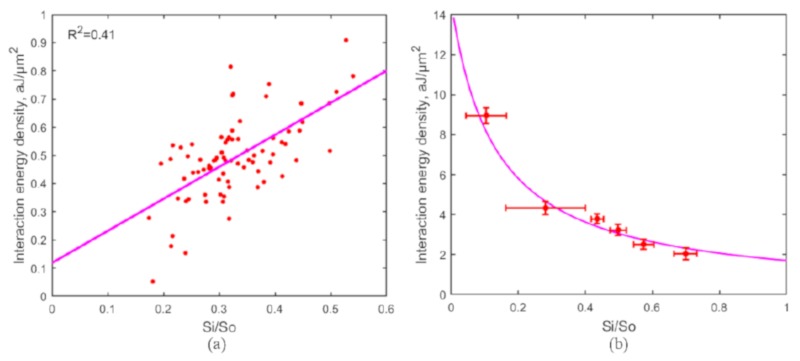
(**a**) RBC interaction energy density measured in aggregation process. 80 pairs of RBCs were measured with different initial overlapping in three independent groups. Experimental data and a linear fitting of the data are shown by red dots and a solid line respectively; (**b**) RBC interaction energy density measured in disaggregation process. 56 pairs of RBCs were measured in three independent groups with six different trapping strength. The reciprocal relationship between the interaction energy density and the relative interaction area is consistent with the “bridging model” prediction.

Among the two co-existent models, the “depletion layer model”, which interprets the RBC clumping as the osmotic pressure caused by a depletion layer of low macromolecule concentration near the interaction surface between two RBCs, has been widely used to describe the aggregation mechanism [29,30]. Aggregation forces of 80 pairs of RBCs were measured with different initial linear overlapping distance (interaction area) in three independent groups to test the repeatability of the results. The corresponding interaction energy densities were calculated and plotted in Figure 4a. The measured aggregation force ($F_{A}$) is very small (in a range of 0.2–4.0 pN), so as the calculated interaction energy density (in a range of 0–0.9 aJ/μm${}^{2}$). The measurement of RBC aggregation was sensitive to a variety of factors including the interference of the measurement environment and operation errors, thus the fluctuations in the measured values are evident. Despite the fluctuations, compared to the “bridging model”, the relationship between the interaction energy density and the relative interaction area measured during RBC aggregation is more consistent with the linear description of the “depletion layer model”. Therefore, a linear fitting was applied to the measured data as shown in Figure 4a. However, as suggested by a recent study [31], the “cross-bridging” mechanism is partly involved in RBC aggregation, thus the relationship may not be accurately described by one single model.

For disaggregation measurement as shown in Figure 3b, after 40 s cell contact at an 80% overlapping area, a constant optical trapping force was applied to slowly drag one RBC away from the other cell until at least one RBC escaped from the trap. The applied optical force was considered to be the disaggregation force ($F_{D}$) to decrease the contact area between the two cells. The interaction energy density reserved in the final interaction area between the two cells at the escaping moment was calculated using Equation (3). The “bridging model” assuming the two RBCs in contact are connected by “bridges” formed by physisorption of macromolecules is widely applied to interpret the intercellular interaction in disaggregation process [30,32]. 56 pairs of RBCs were measured in three independent groups with six different effective trapping power (trapping force) varied from 16.8 mW (3.3 pN) to 36.7 mW (8.0 pN). The obtained interaction energy density varied from 2.0 aJ/μm${}^{2}$ to 9.0 aJ/μm${}^{2}$. A reciprocal relationship between the interaction energy density and the relative interaction area was observed as shown in Figure 4b. According to the ”bridging model”, this relationship follows the expression [32]:

(6) $$D=2k_{B}Tm_{0}b\frac{1}{1+b(S_{i}/S_{0})},$$

where *D* is the interaction energy density and ($S_{i}/S_{0}$) is the relative interaction area; the product of Boltzmann constant and the absolute temperature $k_{B}T$ (4.11 × $10^{-21}$ J) is used as an energy value scaling factor. $m_{0}$ and *b* denoting the cross-bridge density and binding affinity are characteristic parameters of this model, and were calculated to be 1/4260 nm${}^{-2}$ ($m_{0}$) and 7.6 (*b*) in our measurement.

The result indicates that more interaction energy was accumulated within a smaller contact area between the two RBCs during the disaggregation process, which could be explained by the “bridging model” as more “bridges” connecting the two cells need to be broken to separate the two RBCs to a greater extent (to achieve a smaller final contact area). On the other hand, the reciprocal relationship can be explained as the “bridges” were not broken proportionally with the decreasing of contact area, but were sliding towards the conjugated area while remaining attached to both cells during the disaggregation process [2].

### 3.3. Effects of Short-Time Pulsed He–Ne Laser Irradiation on RBC Aggregation

**Figure 5:**
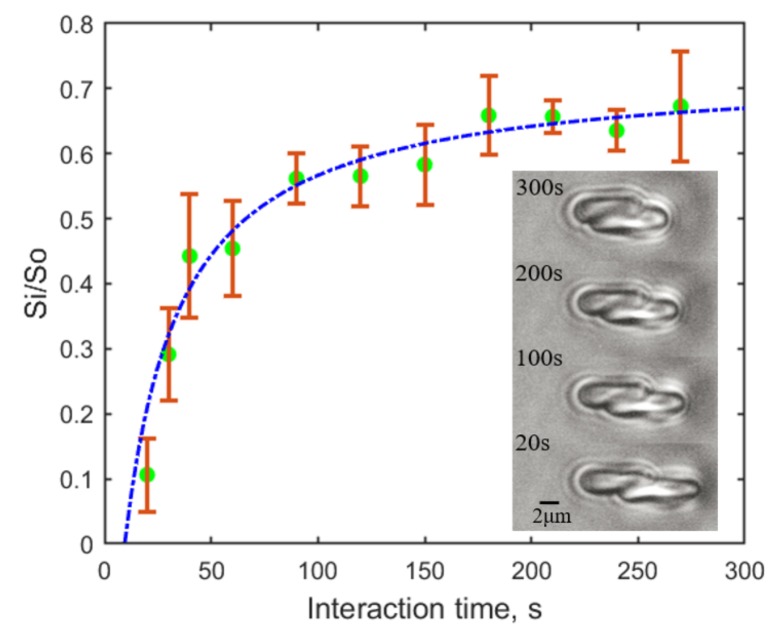
Time-dependence of the final relative adhering area ($S_{i}/S_{0}$) recorded after an enforced separation process by a trapping force of 9.2 pN from an 80% initial contact after different adhering time. 55 pairs of RBCs in total were measured (at least 3 pairs of RBCs for each condition). The green dots show the experimental data with standard deviations and the dashed curve denotes a possible trend of the influence. Microscopic images were captured at the moment of cell escaping, and the time denotes the contact time between the two RBCs before undergoing the disaggregation measurement.

Short irradiation time (up to 300 s) was adopted in our study to minimize thermal effects. As previously reported by us [33], the RBC aggregation dynamics is independent of cell contact time, whereas the measured disaggregation force is strongly influenced by the cell interaction time. With the aforementioned method, different final adhering areas between two RBCs were recorded during the enforced separation by the same trapping force of 9.2 pN after different cell adhering time (0–300 s) at the 80% initial overlapping. The result as shown in Figure 5 indicates that the longer the two RBCs adhere to each other, the harder to separate them from each other. It reveals the time-dependence of the “bridges” formation between two RBCs in the “bridging model”.

**Figure 6:**
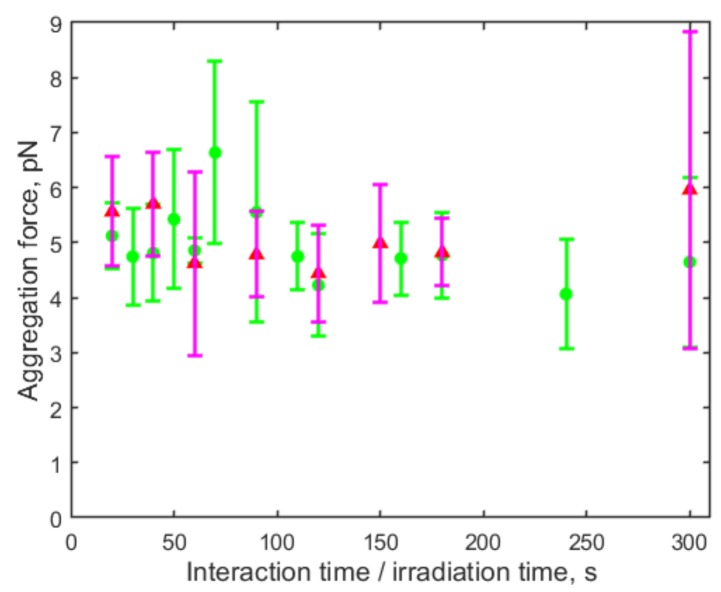
Aggregation forces measured with 32 pairs of RBCs with an initial relative contact area ($S_{i}/S_{0}$) of around 40% over different time of continuous He–Ne laser irradiation (0–300 s) (pink triangles), and the forces measured with 39 pairs of RBCs in the control group (green dots). No significant influence of continuous laser irradiation (within 300 s) on RBC aggregation was observed.

Considering the influence of cell interaction time, the investigation of low-level laser irradiation effects was performed with the RBC aggregation process. An initial relative contact of 40% was formed between two RBCs and the continuous laser irradiation was started at the same time. The irradiation from the He–Ne laser source (power density: 2.21 W/cm${}^{2}$) was directly applied to the trapped RBC rouleau. The RBC aggregation force was measured after different irradiation time. Figure 6 shows the aggregation forces measured in both the irradiated group (pink triangles) and the control group (without laser irradiation) (green dots). The RBC aggregation force was 2.0 ± 0.7 pN in the irradiated group, and 2.0 ± 0.5 pN in the control group. No significant influence of continuous laser irradiation (0–300 s) on RBC aggregation was observed in plasma.

**Figure 7:**
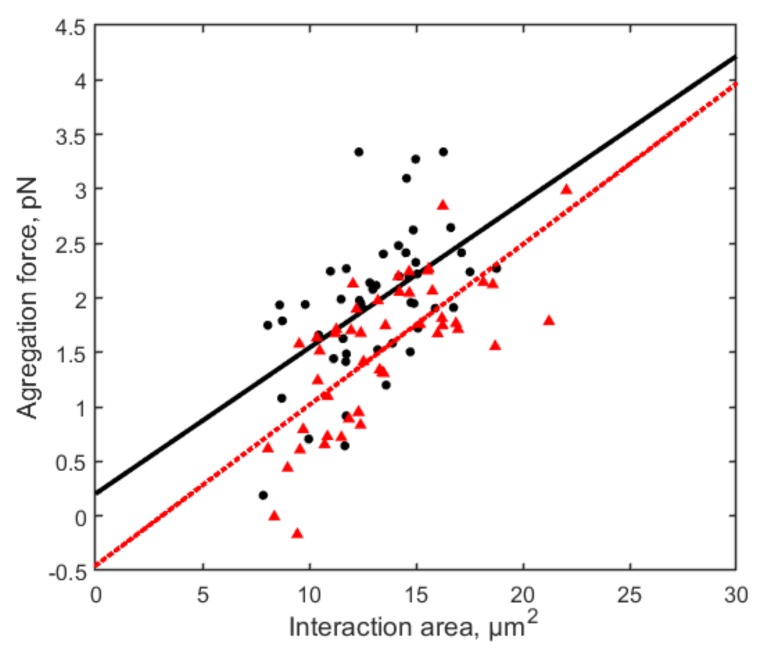
RBC aggregation forces measured with 120 s pulsed He–Ne laser irradiation with pulse frequency of 225 Hz (red triangles, 50 pairs of RBCs) and without irradiation (control group) (black dots, 50 pairs of RBCs). The negative value of aggregation force indicates the two RBCs did not aggregate.

By applying frequency modulation to the continuous-wave He–Ne laser beam with an optical chopper, the effect of pulsed laser irradiation on RBC aggregation was studied. The RBC aggregation force was measured using the aforementioned method under pulsed laser irradiation with frequencies varied from 25 Hz to 10,000 Hz at step size of 25 Hz. The irradiation was applied directly to the trapped RBC rouleau for 60 s, 120 s, and 180 s separately, and the results were compared with the control group (without irradiation). Among all the irradiation conditions, a decrease in RBC aggregation force was observed with 225 Hz pulsed irradiation for 120 s as shown in Figure 7, in which the negative value of aggregation force indicates the two RBCs did not aggregate. Experiments were conducted with 50 pairs of RBCs for control in two groups, and 50 pairs of RBCs for pulsed laser irradiation in three groups. The difference between any of the irradiation groups and any of the control groups is statistically significant (*p* < 0.05) examined by Mann-Whitney U test.

## 4. Discussion

**Figure 8:**
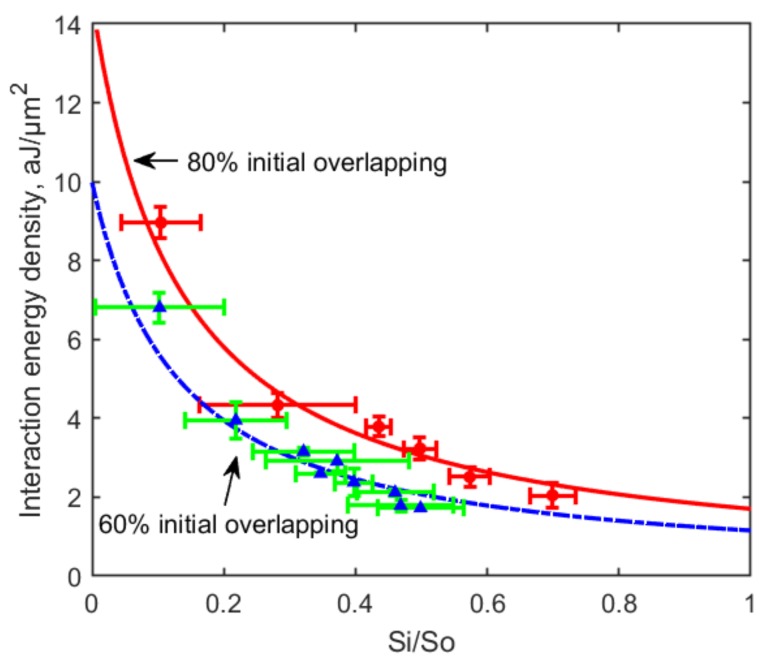
Relationship curves between the RBC disaggregation energy density and relative interaction area measured with 80% (red dots: experimental data, solid line: theory) and 60% (blue triangles: experimental data, dashed line: theory) initial overlapping area. Larger initial overlapping area leads to higher measurable disaggregation interaction energy density between two RBCs.

The RBC dis/aggregation force measured in the present study are consistent with those obtained earlier by Prof A.V. Priezzhev’s group [2,34], whereas the disaggregation energy density is smaller than previously reported by our group [12]. Nevertheless, the obtained characteristic parameters of the “bridging model” (*b* = 7.6 and $m_{0}$ = 1/4260 nm${}^{-2}$) are consistent with our group’s previous study. This can be explained by the dependence of the measured disaggregation energy density on the initial contact area between two RBCs, as shown in Figure 8. It can be seen that larger initial overlapping area leads to higher measurable interaction energy density between two RBCs in the disaggregation measurement. This phenomenon can be interpreted by the “bridging model” as more “bridges” were formed within a larger initial overlapping area, and in order to separate the two cells, more “bridges” needed to be broken compared with a smaller initial overlapping. Thus, stronger disaggregation force and higher interaction energy were measured when the initial overlapping area is larger. A similar observation was also reported by Prof A.V. Priezzhev’s group [35] by measuring the disaggregation forces corresponding to a 20% relative contact area under a 20% or 80% initial overlapping.

Among existing studies on low-level He–Ne laser irradiation effects on blood rheological properties, most results were observed after long-time irradiation (20–130 min) [21,22,23,24], and the thermal effect originating from irradiation energy absorption by hemoglobin was assumed to be the main mechanism. The present study shows that low-level pulsed He–Ne irradiation with pulse frequency of 225 Hz has the potentiality to decreased the RBC aggregation force after a short time of irradiation (120 s). This indicates that other mechanism, such as the modulation of the vibrational state of RBC membrane or membrane-attached proteins might be involved during short-time laser irradiation. Such hypothesis is supported by another study indicating the improvement in RBC deformability after laser irradiation is caused by the detaching of membrane-bound hemoglobin from red cell membrane due to increased vibration energy [21]. However, the systematic evaluation of pulsed laser irradiation effects on RBC interaction dynamics, as well as the detailed mechanism involved in interaction between laser irradiation and RBCs require further in-depth investigation.

As with the majority of studies [12,30,31], the presented results were obtained based on the analysis of the behavior of RBCs taken from one donor, and all measurements were conducted in autologous plasma, thus the results may be specific for the blood of the recruited donor.

## 5. Conclusions

Red blood cell (dis)aggregation properties were investigated in detail by optical tweezers. A linear and a reciprocal dependence of the interaction energy density on the relative interaction area between two RBCs in autologous plasma were obtained in aggregation and disaggregation process, respectively. The interaction energy measured in RBC disaggregation is influenced by the measurement conditions (e.g., initial cell overlapping area and contact time) and has different orders of magnitude than that measured in RBC aggregation. In addition, up to 300 s continuous He–Ne laser irradiation (power density: 2.21 W/cm${}^{2}$) showed no influence on RBC aggregation, whereas the RBC aggregation force decreased after 120 s irradiation by the same laser with pulse modulation of 225 Hz. The observation indicates the potentiality to modulate RBC aggregation properties by short-time laser irradiation. Our study provides new insights into the investigation of the dynamic interaction between RBCs, and the evaluation of the regulating effects of low-level laser irradiation on RBC interaction properties.
